# Heat Shock Protein 27 Injection Leads to Caspase Activation in the Visual Pathway and Retinal T-Cell Response

**DOI:** 10.3390/ijms22020513

**Published:** 2021-01-06

**Authors:** Pia Grotegut, Philipp Johannes Hoerdemann, Sabrina Reinehr, Nupur Gupta, H. Burkhard Dick, Stephanie C. Joachim

**Affiliations:** Experimental Eye Research Institute, University Eye Hospital, Ruhr-University Bochum, In der Schornau 23-25, 44892 Bochum, Germany; pia.grotegut@rub.de (P.G.); Philipp.Hoerdemann@rub.de (P.J.H.); sabrina.reinehr@rub.de (S.R.); nupur.gupta@uni-duesseldorf.de (N.G.); Burkhard.Dick@kk-bochum.de (H.B.D.)

**Keywords:** glaucoma, heat shock protein 27 (HSP27), retinal ganglion cell degeneration, apoptosis, T-cells, microglia, NFκ-B

## Abstract

Heat shock protein 27 (HSP27) is one of the small molecular chaperones and is involved in many cell mechanisms. Besides the known protective and helpful functions of intracellular HSP27, very little is known about the mode of action of extracellular HSP27. In a previous study, we showed that intravitreal injection of HSP27 led to neuronal damage in the retina and optic nerve after 21 days. However, it was not clear which degenerative signaling pathways were induced by the injection. For this reason, the pathological mechanisms of intravitreal HSP27 injection after 14 days were investigated. Histological and RT-qPCR analyses revealed an increase in endogenous HSP27 in the retina and an activation of components of the intrinsic and extrinsic apoptosis pathway. In addition, an increase in nucleus factor-kappa-light-chain-enhancer of activated B cells (NFκB), as well as of microglia/macrophages and T-cells could be observed. In the optic nerve, however, only an increased apoptosis rate was detectable. Therefore, the activation of caspases and the induction of an incipient immune response seem to be the main triggers for retinal degeneration in this intravitreal HSP27 model.

## 1. Introduction

Heat shock proteins (HSPs) are molecular chaperones, and they are upregulated in response to physiological and environmental stressors [1]. In general, HSPs are attributed protective functions, such as protein stabilization and reduction of apoptosis. HSPs can be classified into different groups according to their molecular weight [2]. Larger proteins, such as HSP70 and HSP60 chaperones, are found in the cytosol, endoplasmic reticulum, mitochondria, and the cell nucleus, as well as in the extracellular environment [3,4].

One of the smaller HSPs is HSP27, also known as HSP25 or heat shock protein beta-1 (HspB1). It is ubiquitously expressed and is involved in various biological functions [5]. Intracellularly, HSP27 can inhibit the cytochrome C-mediated activation of caspases in the cytosol [6]. Furthermore, HSP27 can indirectly prevent the activation of BCL-2-associated X protein (Bax) [7]. Extracellularly, HSP27 can serve as a signal molecule and bind to membrane receptors, such as toll-like receptors (TLRs). In mouse coronary endothelial cells, extracellular HSP27 interaction between TLR2 and TLR4 was observed, which then led to nucleus factor-kappa-light-chain-enhancer of activated B cells (NFκB) phosphorylation [8]. In addition, the treatment of human macrophages with recombinant HSP27 led to increased NFκB transcriptional activity. Treatment of macrophages with HSP27 also resulted in increased expression of a variety of genes, including proinflammatory factor interleukin (IL)-1β and tumor necrosis factor α. Increased anti-inflammatory factors, such as IL-10 and GM-CSF, were also detected at both mRNA and protein levels [9]. This suggests that the mode of action of HSP27 may depend on the localization.

In recent years, studies have been carried out to analyze the relationship among neuronal diseases and HSPs. One of these diseases is glaucoma, whose main feature is the destruction of retinal ganglion cells (RGCs) and their axons [10]. Studies in donor eyes of glaucoma patients demonstrated an increased expression of HSP27 in the nerve fiber layer, RGCs, retinal vessels, and optic nerve heads [11]. Furthermore, increased HSP27 levels in the retina were also detected in various glaucoma models [12]. For example, laser photocoagulation of episcleral and limbal veins induced glaucoma in an animal model. It was observed that the expression of HSP27 was increased in RGCs and retinal astrocytes of laser treated eyes [13]. In the same publication, it was assumed that prolonged HSP27 expression is a cell-specific phenomenon and could serve as a warning sign for neuronal damage. Tezel et al. postulated that the increased expression of HSPs in glaucomatous eyes may initially serve to protect cells from further destruction and facilitate repair. However, the authors also assumed that long-lasting HSP expression could recruit immune responses that contribute to disease progression [11]. Lately, some investigations have also demonstrated that an upregulation of HSPs might be problematic and cause neuronal loss. For example, Chen et al. noted that HSP27 upregulation induced an immune mediated neural damage through activating HSP-specific CD4^+^ T-cell response [14]. In animal models, it was observed that systemic administration of HSP27 led to glaucoma-like damage [15,16]. Recently, we also demonstrated that intravitreal injection of HSP27 induced a degeneration of RGCs and the optic nerve neurofilament after 21 days [17]. These results suggest that HSP27 may be involved in degenerative mechanisms. However, it is not clear which pathological mechanisms are activated by the increased extracellular HSP27 concentration.

Therefore, this study deals with the possible pathological mechanisms induced through intravitreal HSP27 injection, with a focus on caspase and immunological response in retina and optic nerve.

## 2. Results

### 2.1. Increased Heat Shock Protein Levels on Protein and Gene Level

**Figure 1:**
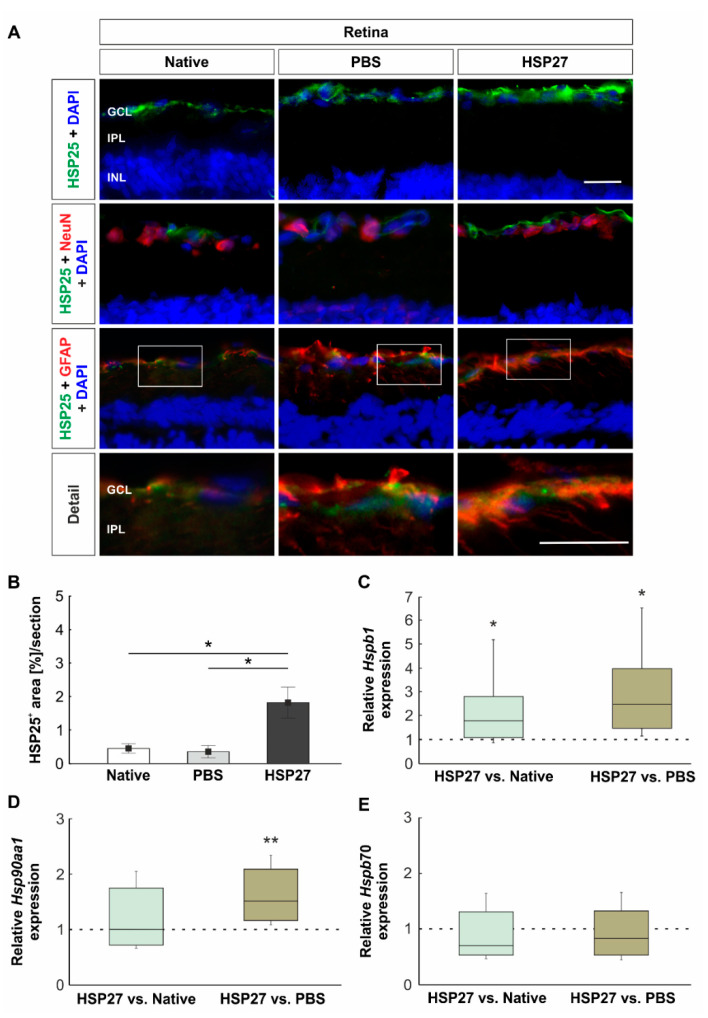
Localization and expression level of heat shock proteins. (**A**) Heat shock protein 25 (HSP25, green), astrocytes (GFAP, red), neuronal cells (NeuN, red), and cell nuclei (DAPI, blue) were stained on retinal cross-sections. The detail view revealed that HSP25 signals were rather colocalized with GFAP signals than with NeuN signals (detail). (**B**) The HSP25 signal was strongly increased in the heat shock protein 27 (HSP27) group as compared with the native (*p* = 0.017) and PBS group (*p* = 0.014). (**C**) Compared to controls, a significant increase in *Hspb1* mRNA expression was measured in HSP27 treated retinae (HSP27 vs. native: *p* = 0.040 and HSP27 vs. PBS: *p* = 0.010). (**D**) Via RT-qPCR, a significant increase in *Hsp90aa1* mRNA expression was also observed in the HSP27 group as compared with the PBS group (*p* = 0.002), but not compared with the native group. (**E**) In contrast, the relative mRNA expression of *Hspb70* was similar in all groups. Scale bars = 20 μm. GCL, ganglion cell layer; IPL, inner plexiform layer; INL, inner nuclear layer. * *p* < 0.05 and ** *p* < 0.01. Immunohistology = mean ± SEM, RT-qPCR = median ± quartile + minimum/ maximum. Dashed line represents the relative expression level of the control group.

To investigate the level and localization of HSP27, an antibody against HSP25, known as the rodent HSP27, was used (Figure 1A). After HSP25 staining, an area evaluation of the HSP25^+^ signal was performed. The native (0.5 ± 0.1%) and the PBS (0.4 ± 0.2%) groups showed very similar HSP25^+^ area size (*p* = 1.0). In contrast, the HSP25^+^ staining area in the HSP27 group (1.8 ± 0.5%) was increased (HSP27 vs. native: *p* = 0.017 and HSP27 vs. PBS: *p* = 0.014; Figure 1B).

To identify the localization of HSP25, it was stained in combination with a marker against neuronal cells (NeuN) and astrocytes (GFAP). According to these overview stains, we noted that HSP25 was mainly located in the ganglion cell layer (GCL). However, a stronger colocalization between HSP25 and GFAP was observed, suggesting that HSP25 was released by astrocytes (Figure 1A, detail).

In order to evaluate the HSP27 (*Hspb1*), HSP90 (*Hsp90aa1*), and HSP70 (*Hspb70*) expression on mRNA level, RT-qPCR analyses were performed. Compared to controls, a significant upregulation of relative *Hspb1* expression was detected in the HSP27 group (HSP27 vs. native: 1.8-fold expression, *p* = 0.040 and HSP27 vs. PBS: 2.5-fold expression, *p* = 0.010, Figure 1C). In contrast, the relative expression of *Hsp90aa1* in the HSP27 group was only increased as compared with the PBS group (1.5-fold expression, *p* = 0.002, Figure 1D). No differences in the relative expression of *Hsp90aa1* could be measured between the HSP27 and native group (1.0-fold expression, *p* = 0.990). Similar results were obtained for the relative expression of *Hspb70*. No differences were found between the HSP27 group and the native group (0.7-fold expression, *p* = 0.108), as well as the PBS group (0.8-fold expression, *p* = 0.377, Figure 1E).

### 2.2. Unaltered Number of Retinal Ganglion Cells but Increased Apoptosis Rate

**Figure 2:**
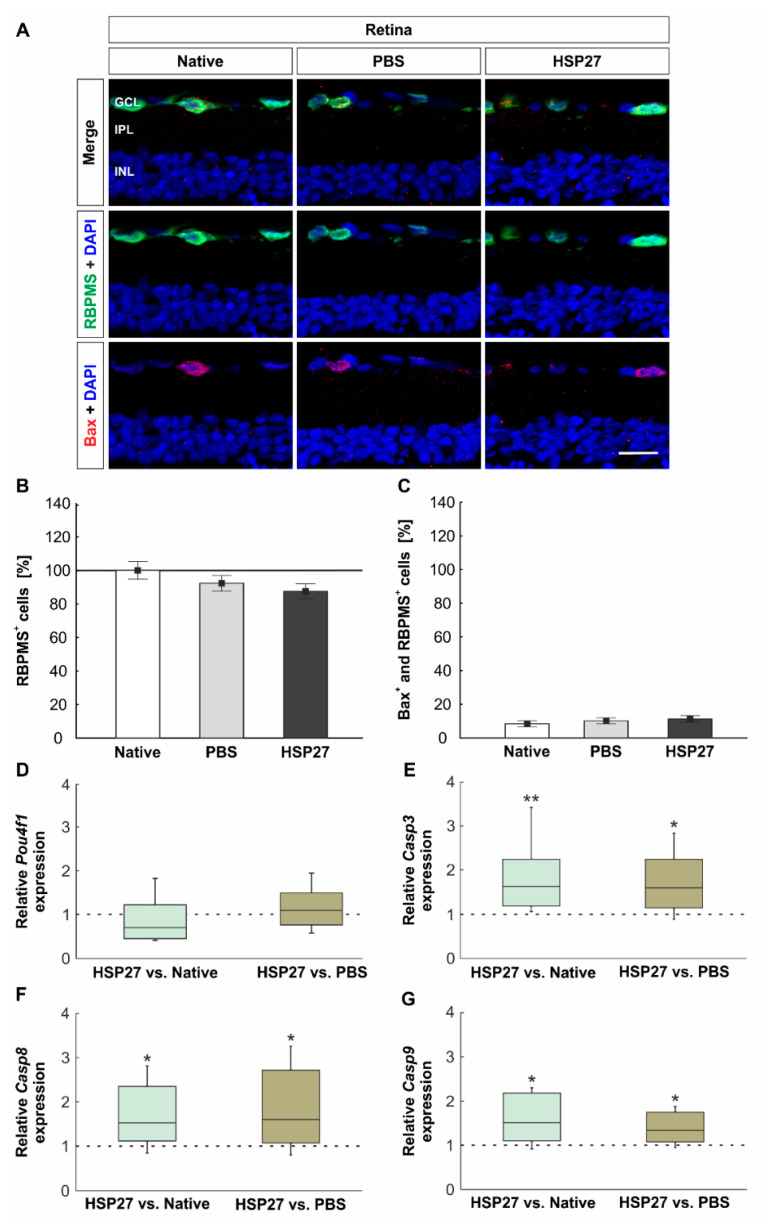
Constant number of retinal ganglion cells but increased apoptosis rate. (**A**) Retinal ganglion cells (RGCs) in the retina were marked with RBPMS (green), apoptotic RGCs with Bax (red), and cell nuclei with DAPI (blue). (**B**) RBPMS cell count revealed no differences among the HSP27, the PBS, and the native group. (**C**) Bax^+^ cell counts also demonstrated no differences among the groups. (**D**) The expression level of *Pou4f1* (marker for RGC), investigated by RT-qPCR, was similar in the groups. (**E**) The relative expression of *Casp3* mRNA was significantly increased in the HSP27 group as compared with the native group (*p* = 0.003) and the PBS group (*p* = 0.042). (**F**) Via RT-qPCR, an increase in *Casp8* mRNA expression was detected in HSP27 retinae as compared with both control groups (native: *p* = 0.039 and PBS: *p* = 0.046). (**G**) The mRNA expression of *Casp9* was also increased in HSP27 animals as compared with the native group (*p* = 0.024) and PBS group (*p* = 0.029). Scale bar = 20 µm. GCL, ganglion cell layer; IPL, inner plexiform layer; INL, inner nuclear layer. * *p* < 0.05 and ** *p* < 0.01. Immunohistology = mean ± SEM, RT-qPCR = median ± quartile + minimum/maximum. Dashed line represents the relative expression level of the control group, solid line represents 100%.

To analyze neuronal degeneration, we quantified the number of RGCs and the number of RGCs in apoptotic stage using RBPMS and Bax (Figure 2A). The number of RGCs was almost equal in the HSP27 group (96.6 ± 5.6%), the PBS (98.2 ± 6.0%, *p* = 0.991), and the native group (100.0 ± 12.4%, *p* = 0.959, Figure 2B). Both control groups also demonstrated comparable RGC counts (native vs. PBS: *p* = 0.987). Regarding the number of Bax^+^ and RBPMS^+^ cells, no differences could be observed in the HSP27 group (11.3 ± 1.9%) when compared with both controls (PBS 10.2 ± 1.8%, *p* = 0.887 and native 8.4 ± 1.0%, *p* = 0.450). Similar numbers of apoptotic RGCs were also observed in the PBS and the native group (*p* = 0.730, Figure 2C).

In order to evaluate the mRNA level, expression of *Pou4f1*, a marker for RGCs, was analyzed via RT-qPCR. The investigation showed no differences in the relative expression of *Pou4f1* between the groups (HSP27 vs. native: 0.7-fold expression, *p* = 0.139 and HSP27 vs. PBS: 1.1-fold expression, *p* = 0.639, Figure 2D). In contrast, the analysis of mRNA expression of caspase 3 (*Casp3*), 8 (*Casp8*), and 9 (*Casp9*) demonstrated strong changes in HSP27 retinae. Regarding the relative *Casp3* expression, a significant upregulation could be measured in the HSP27 group (HSP27 vs. native: 1.6-fold expression, *p* = 0.003 and HSP27 vs. PBS: 1.6-fold expression, *p* = 0.042, Figure 2E). Investigation of the relative *Casp8* mRNA revealed a significant elevation in the HSP27 group in relation to the native (1.5-fold expression, *p* = 0.039) and the PBS group (1.6-fold expression, *p* = 0.046, Figure 2F). The relative expression of *Casp9* was also increased in HSP27 retinae as compared with the native group (1.5-fold expression, *p* = 0.024) and the PBS group (1.3-fold expression, *p* = 0.029, Figure 2G).

### 2.3. HSP27 Could Activate the NFκB Signal Pathway

**Figure 3:**
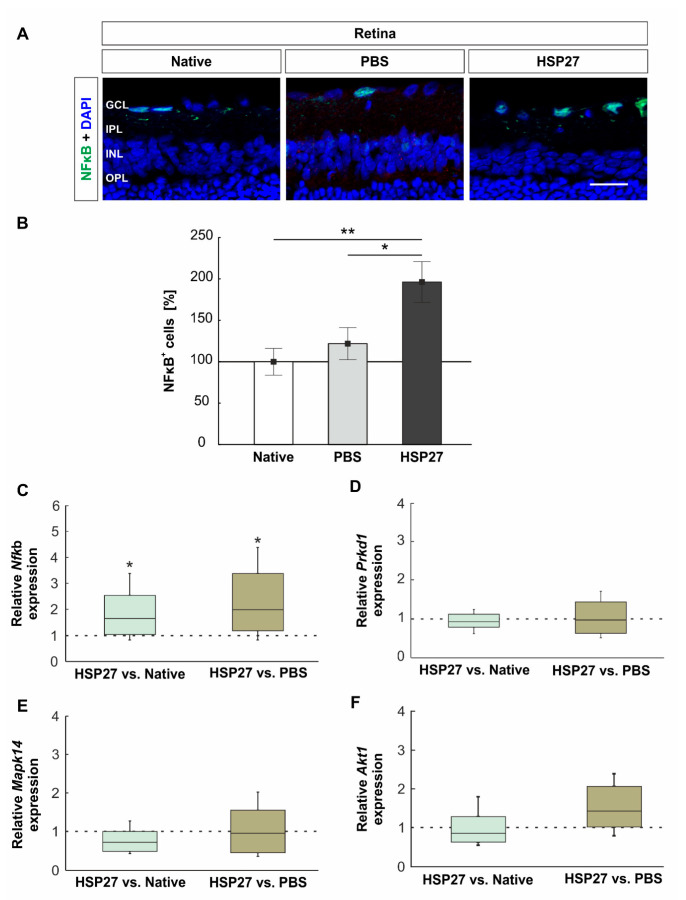
Increased NFκB signals after HSP27 injection. (**A**) Nucleus factor-kappa-light-chain-enhancer of activated B cells (NFκB, green) and DAPI (blue) were stained on retinal cross-sections. (**B**) More NFκB signals were noted in the HSP27 group than in the native (*p* = 0.008) and PBS group (*p* = 0.043). (**C**) The relative *NfκB* mRNA expression level in the HSP27 group was increased as compared with the native group (*p* = 0.049) and PBS group (*p* = 0.017). (**D**) Regarding *Prkd1* mRNA expression, no differences were noted. (**E**) The relative *Mapk14* mRNA expression level in HSP27 retinae was similar as compared with both control groups. (**F**) Analysis of relative *Akt1* mRNA expression revealed no differences among the groups. Scale bar = 20 µm. GCL, ganglion cell layer; IPL, inner plexiform layer; INL, inner nuclear layer; OPL, outer plexiform layer. * *p* < 0.05 and ** *p* < 0.01. Immunohistology = mean ± SEM, RT-qPCR = median ± quartile + minimum/ maximum. Dashed line represents the relative expression level of the control group, solid line represents 100%.

To investigate further degenerative mechanisms, the NFκB signal was investigated by immunohistochemistry (Figure 3A) and the relative mRNA expression of NFκB (*Nfκ*b), protein kinase D (*Prkd1*), p38 (*Mapk14*), and serine/threonine kinase 1 (*Akt1*) was determined via RT-qPCR (Figure 3). Fourteen days after intravitreal HSP27 injection, more NFκB^+^ cells in the GCL were noted in this group (196.3 ± 24.6%) when compared with native (100.0 ± 16.2%, *p* = 0.008) and PBS retinae (121.9 ± 19.3%, *p* = 0.043). Both controls had similar NFκB^+^ cell counts (*p* = 0.731, Figure 3B).

The RT-qPCR results were in accordance with histological data. The HSP27 group displayed a significantly higher expression of *Nfκ*b mRNA expression than the native (1.7-fold expression, *p* = 0.049) and PBS group (2.0-fold expression, *p* = 0.017; Figure 3C). In contrast, the relative expression of *Prkd1* was comparable in all three groups (HSP27 vs. native: 0.9-fold expression, *p* = 0.455 and HSP27 vs. PBS: 1.0-fold expression, *p* = 0.905, Figure 3D). The relative *Mapk14* mRNA expression level in HSP27 retinae was also similar to both control groups (HSP27 vs. native: 0.7-fold expression, *p* = 0.103 and HSP27 vs. PBS: 1.0-fold expression, *p* = 0.859, Figure 3E). Likewise, the analysis of relative *Akt1* mRNA expression revealed no differences between the groups (HSP27 vs. native: 0.8-fold expression, *p* = 0.378 and HSP27 vs. PBS: 1.4-fold expression, *p* = 0.444, Figure 3F).

### 2.4. Increased Microglia/Macrophages and T-Cell Number in Retinae through HSP27

**Figure 4:**
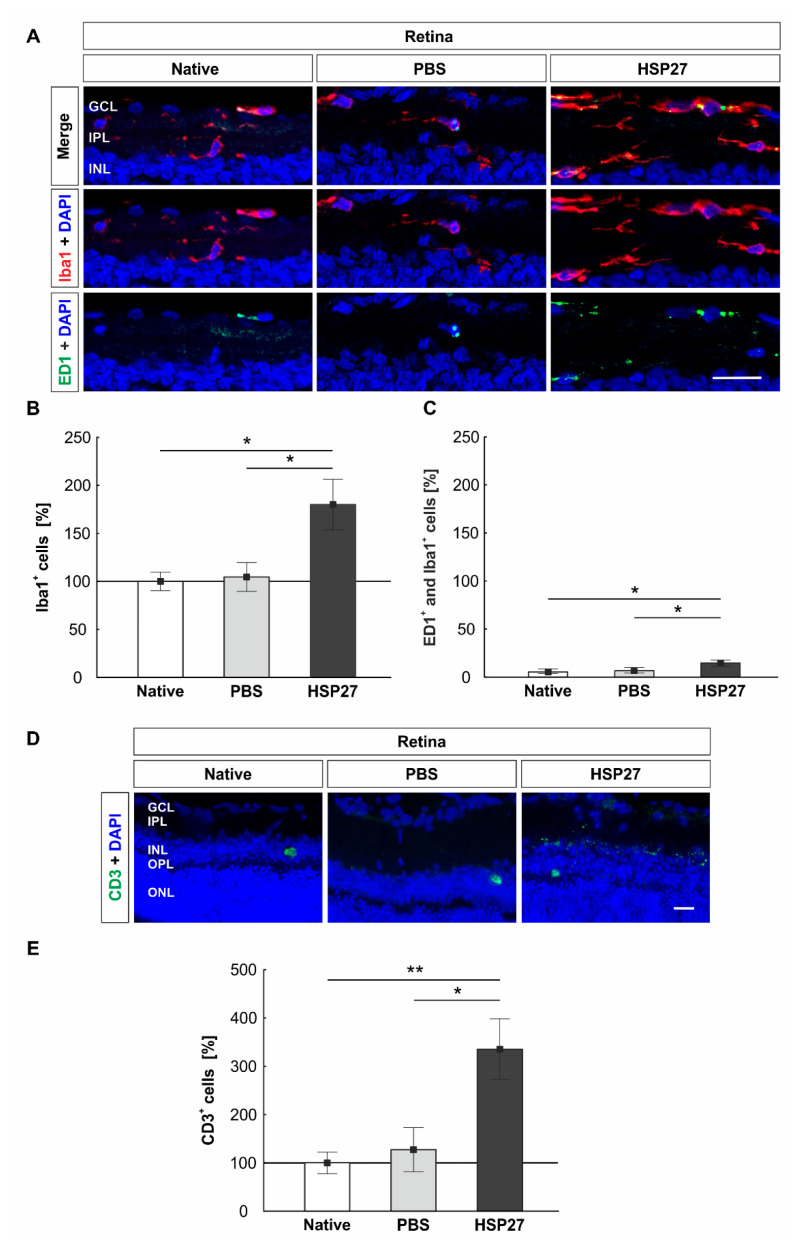
Intravitreal HSP27 injection induced microglia/macrophage and T-Cell response. (**A**) Microglia/macrophages (Iba1, red) in an active state (ED1, green, arrows) were marked on retinal cross-sections. DAPI (blue) was used to visualize cell nuclei. (**B**) HSP27 increased the number of microglia (native, *p* = 0.015 and PBS, *p* = 0.022). (**C**) In HSP27 retinae, a higher number of ED1^+^ and Iba1^+^ cells was noted as compared with both controls (native: *p* = 0.013 and PBS: *p* = 0.034). (**D**) CD3^+^ T-cells (green) and cell nuclei (DAPI, blue) were stained in retinae. (**E**) More T-cells were counted in HSP27 retinae than in both controls (native: *p* = 0.008 and PBS: *p* = 0.015). Scale bar = 20 µm. GCL, ganglion cell layer; IPL, inner plexiform layer; INL, inner nuclear layer; OPL, outer plexiform layer; ONL, outer nuclear layer. * *p* < 0.05 and ** *p* < 0.01. Immunohistology = mean ± SEM. Solid line represents 100% cells.

We used the antibody Iba1, which labels the whole microglia/macrophage population [18], and ED1, which labels activated microglia/macrophages, for staining (Figure 4A). At day 14, the number of microglia/macrophages in the HSP27 group (180.0 ± 26.3%) was increased as compared with the native (100.0 ± 9.7%, *p* = 0.015) and the PBS group (104.6 ± 15.1%, *p* = 0.022, Figure 4B). The native and the PBS group showed no differences (*p* = 0.982). The number of active microglia/macrophages doubled in the HSP27 group (14.6 ± 8.6%) as compared with the native group (5.3 ± 4.8%, *p* = 0.013) and the PBS group (6.7 ± 2.8%, *p* = 0.034). Both controls displayed similar numbers of ED1^+^ and Iba1^+^ cells (*p* = 0.988, Figure 4C).

Recently, it was speculated whether the increased concentration of HSP27 in glaucoma patients leads to a specific immune response. First investigations showed that high HSP27 concentrations caused a reaction of the T-cells [14]. Hence, retinal T-cells were stained with the marker CD3 (Figure 4D). Overall, only a few T-cells could be detected in the entire retina. However, cell counts revealed differences among the groups. At day 14, the HSP27 group revealed more T-cells (335.3 ± 62.9%) than the native group (100.0 ± 22.1%, *p* = 0.008) or the PBS one (127.3 ± 45.9%, *p* = 0.015, Figure 4E). No difference was noted between the PBS and native group (*p* = 0.919).

### 2.5. Unaltered HSP25 and NFκB Signals in Optic Nerves

**Figure 5:**
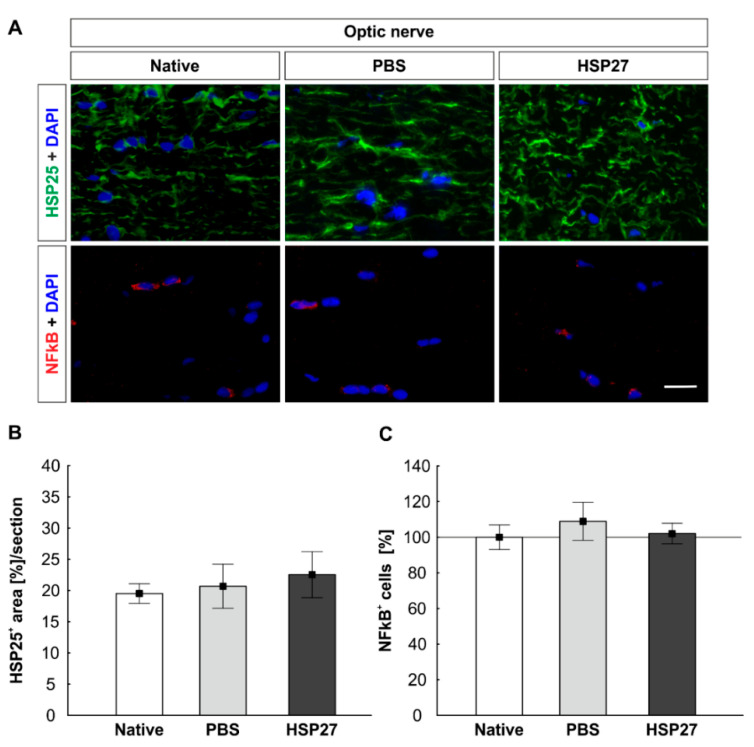
Constant HSP25 expression and unchanged NFκB signal in the optic nerve. (**A**) HSP25 (green), NFκB (red), and cell nuclei (DAPI, blue) were labeled on optic nerve longitudinal sections. (**B**) The evaluation of the percentage area of the HSP25 signals showed no differences among the three groups. (**C**) The counting of the NFkB^+^ signals also showed no differences among the groups. Scale bar = 20 µm. Immunohistology = mean ± SEM. Solid line represents 100% cells.

The HSP27 signal was also histologically examined with an antibody against the rodent homologue HSP25 in the optic nerve (Figure 5A). Interestingly, the evaluation of the percentage area of labelled HSP25 did not show any significant differences among the HSP27 optic nerves (22.5 ± 3.7%), the native animals (19.5 ± 1.6%, *p* = 0.769), and the PBS group (20.7 ± 3.5%, *p* = 0.904). In addition, between the PBS and native groups no differences were noted (*p* = 0.962, Figure 5B).

In addition, NFκB was histologically examined in the optic nerve, since it is an important transcription factor (Figure 5A). The number of NFκB^+^ cells was equal in all three groups (native: 100.0 ± 6.9%, PBS: 108.9 ± 10.7%, and HSP27: 102.1 ± 5.8%, HSP27 vs. native: *p* = 0.982, HSP27 vs. PBS: *p* = 0.822, and PBS vs. native, *p* = 0.719, Figure 5C).

### 2.6. Increased Apoptosis Rate in the Optic Nerve

**Figure 6:**
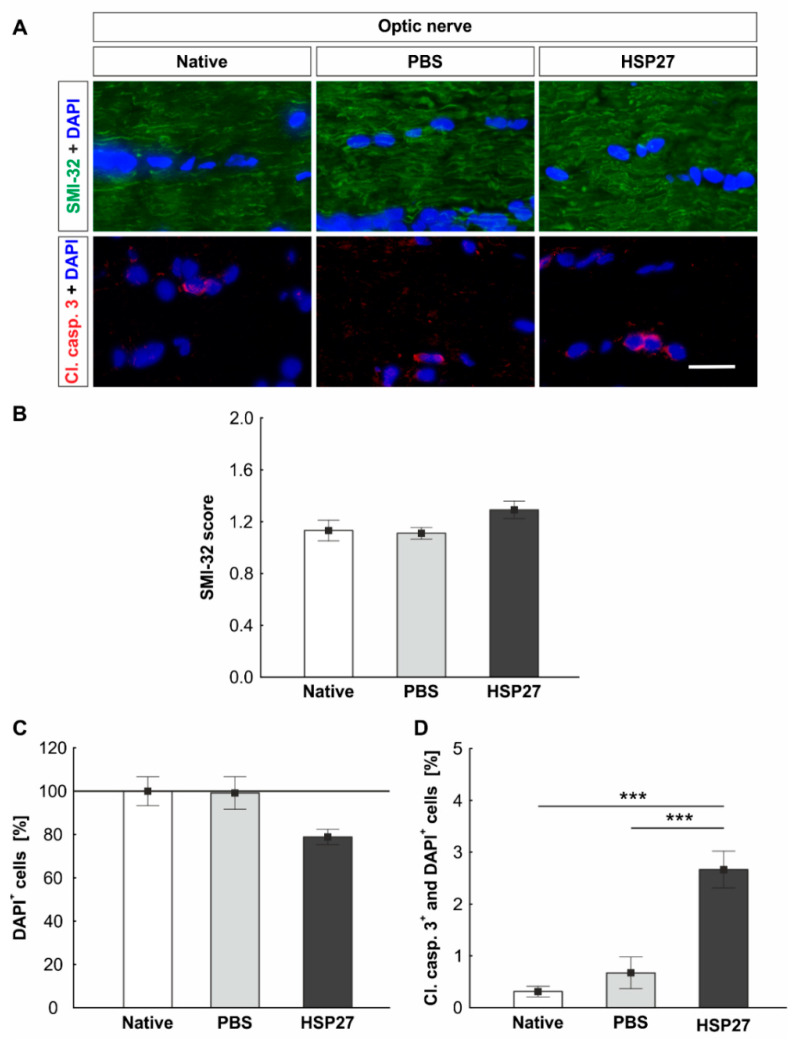
The optic nerves show an increased rate of apoptosis after HSP27 injection. (**A**) The antibody against SMI-32 (green) was applied to identify the neurofilaments in the optic nerve. Cleaved caspase 3 (red) was used to detect apoptotic cells and cell nuclei were stained with DAPI (blue). (**B**) Fourteen days after HSP27 injection, no effects on the neurofilament were noted via SMI-32 scoring. (**C**) The number of DAPI^+^ cell nuclei did not change significantly. (**D**) The cleaved caspase 3 cell count revealed an increase of apoptotic cells in the HSP27 group as compared with the native group (*p* < 0.001) and PBS group (*p* < 0.001). Scale bar = 20 µm. *** *p* < 0.001 and immunohistology = mean ± SEM. Solid line represents 100% cells.

The neurofilament of optic nerves was stained with SMI-32 (Figure 6A). No differences among the PBS group (1.1 ± 0.1 mean score), the HSP27 group (1.3 ± 0.2 mean score), and the native group (1.1 ± 0.1 mean score) were detected regarding the SMI-32 score (PBS vs. native: *p* = 0.972, HSP27 vs. native: *p* = 0.223, and HSP27 vs. PBS: *p* = 0.153, Figure 6B).

Apoptotic cells were investigated in the optic nerve via immunohistochemistry using a cleaved caspase 3 antibody (Figure 6A). To determine the number of degenerated cells in the optic nerve, DAPI^+^ cells, as well as cleaved caspase 3^+^ and DAPI^+^ cells, were counted. The number of nuclei was slightly, but not significantly reduced in the optic nerves of the HSP27 group (78.9 ± 3.6%) as compared with the native group (100.0 ± 6.7%, *p* = 0.061) and the PBS group (99.2 ± 7.5%, *p* = 0.074). No difference could be seen between the native and the PBS groups (*p* = 0.995, Figure 6C). In contrast, the number of apoptotic cells was significantly higher in the HSP27 group (2.7 ± 0.4%) as compared with the native (0.3 ± 0.1%, *p* < 0.001) and the PBS optic nerves (0.7 ± 0.3%, *p* < 0.001). Native and PBS optic nerves demonstrated similar, low numbers of apoptotic cells (*p* = 0.633, Figure 6D).

### 2.7. Constant Number of Microglia/Macrophages and T-Cells in the Optic Nerves

**Figure 7:**
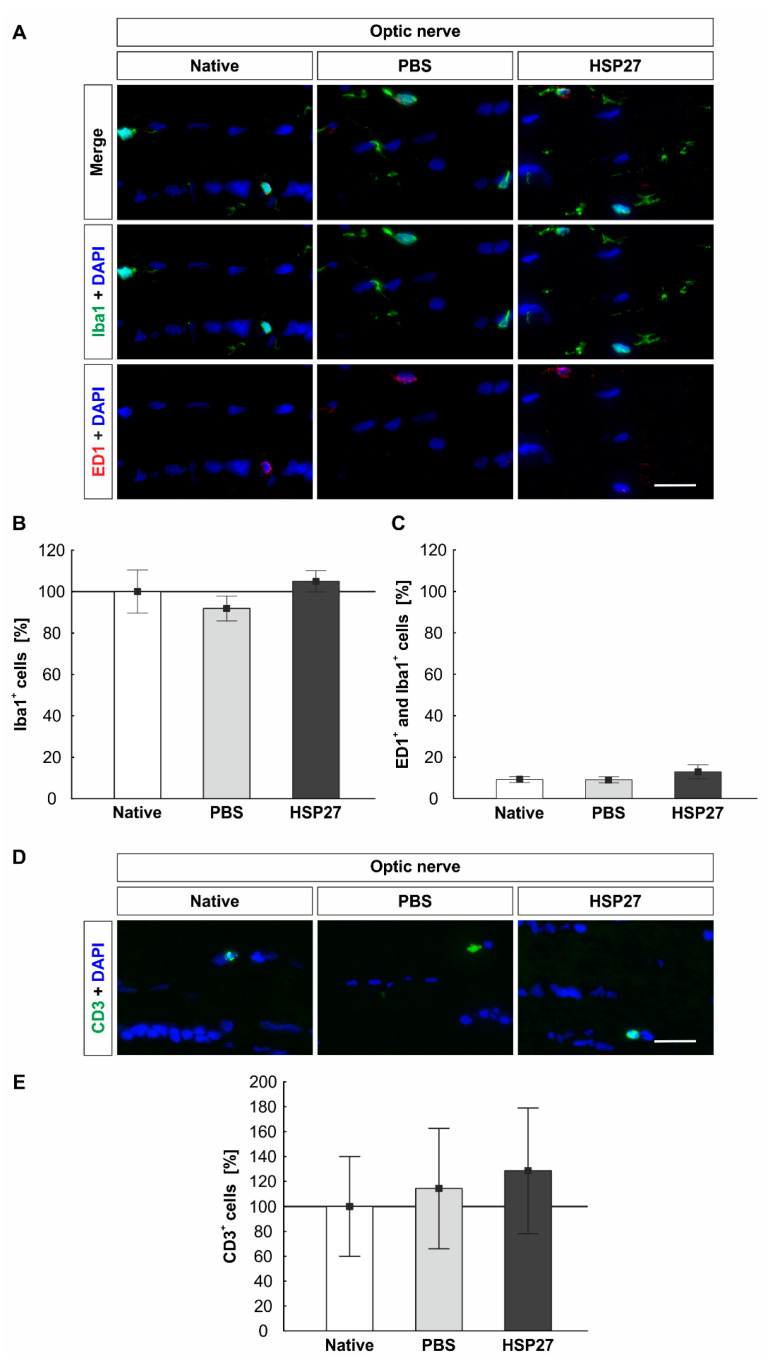
Constant number of microglia/macrophages and T-cells in the optic nerve. (**A**) Microglia/macrophages (Iba1, green) and microglia/macrophages in active state (ED1, red), as well as nuclei (DAPI, blue), were marked in the optic nerve. (**B**) The number of microglia/macrophages was comparable in all groups. (**C**) Likewise, no differences among the native group, the PBS group, and the HSP27 group could be observed regarding to the number of activated microglia/macrophages. (**D**) T-cells (CD3, green) and nuclei (DAPI, blue) were stained in the optic nerve. (**E**) Evaluation of the CD3^+^ signals showed no significant differences among the groups. Scale bar = 20 µm. Immunohistology = mean ± SEM. Solid line represents 100% cells.

Microglia/macrophages, as well as activated microglia/macrophages, were also visualized in the optic nerve (Figure 7A). The counting of the Iba1^+^ signals showed no differences among the groups (native: 100.0 ± 10.4%, PBS: 91.8 ± 6.1%, HSP27:105.0 ± 5.2%, HSP27 vs. native: *p* = 0.889, HSP27 vs. PBS: *p* = 0.454, and PBS vs. native: *p* = 0.732, Figure 7B). The number of ED1^+^ and Iba1^+^ signals was also statistically equal in the native (9.1 ± 1.4%), the PBS (9.1 ± 1.4%), and the HSP27 (12.9 ± 3.3%) groups (PBS vs. native: *p* = 1.000, HSP27 vs. native: *p* = 0.469, and HSP27 vs. PBS: *p* = 0.459, Figure 7C).

The number of CD3^+^ T-cells was examined in the optic nerve (Figure 7D). Only very few positive signals could be detected. Counting of CD3^+^ signals revealed no differences among the native group (100.0 ± 40.0%), the PBS group (114.3 ± 48.3%), and the HSP27 group (128.6 ± 50.4%, PBS vs. native: *p* = 0.974, HSP27 vs. native: *p* = 0.902, and HSP27 vs. PBS: *p* = 0.974, Figure 7E).

## 3. Discussion

HSP27 is a small chaperone and is considered to be a protective protein in the intracellular environment. The extracellular mode of action of HSP27 has only been investigated in a few cases. In this study, we demonstrated that extracellular HSP27 induced degenerative signaling pathways, including caspase activation, hence, leading to cell death.

### 3.1. Endogenous HSP as a Response to Stress

Fourteen days after intravitreal HSP27 injection, the HSP response in retinae and optic nerves was examined. Many neuronal diseases result in an increased intracellular expression of HSPs. Since these diseases are often accompanied by an accumulation of insoluble protein aggregates or amyloid fibrils in neurons or glial cells, it is assumed that HSP27 is increasingly expressed to reduce this accumulation. For example, neurons of Alzheimer’s patients display a colocalization of Lewy’s bodies and HSP27 [19]. Many other studies also point to a stress-dependent induction of HSP27 [20,21,22,23]. Therefore, the increased protein and mRNA concentration of endogenous HSP27, in our study, suggests that intravitreal HSP27 injection exerted a form of stress on retinal cells. The reaction appeared to be concentrated in the GCL, as elevated HSP25^+^ signals were detected histologically mainly in this layer. This was consistent with other studies that found elevated levels of HSP27 in the GCL of rodents with glaucomatous damage, optic nerve injury, or induced retinal damage [12,13,24]. After closer analysis, the astrocytes appeared to be associated with HSP27 in our study. This is in accordance with investigations in eyes from an experimental rat glaucoma model and the DBA/2J mice. There, it was shown that increased HSP27 and phosphorylated HSP27 occurred mainly in glial cells [25]. Considering gene level, the expression of HSP27 was enhanced while the analysis of HSP70 presented no differences among the groups. Furthermore, the expression of HSP90 seemed to be increased as well. Therefore, intravitreal injection of HSP27 seems to induce endogenous expression of HSPs in the retina. Possibly this is a response to the degenerative signaling pathways. Interestingly, we could not detect an increased HSP27 expression in the optic nerve. Therefore, the optic nerve does not seem to be exposed to the same stress response from HSP27 injection.

### 3.2. Activation of Caspase-Dependent Cell Death

In a previous study, it was demonstrated that intravitreal HSP27 injection led to a loss of retinal ganglion and amacrine cells after 21 days [17]. However, no degenerative signaling pathways or apoptotic signals were identified at that time. For this reason, RGCs and apoptotic signaling pathways were studied at an earlier stage, i.e., 14 days after injection, in the current study. Interestingly, after 14 days, no effects on RGCs were detected, cell counts were comparable in the three groups. This is in contrast to intravitreal injections of other proteins. For example, the injection of S100B induced the loss of RGCs and amacrine cells after 14 days [26]. Likewise, the intravitreal injection of N-methyl-D-aspartate (NMDA) or glutamate caused a rapid degeneration of the RGCs already after 2–3 days [27,28]. The reason for the different times of degeneration lies in the mode of action of the injected substances. Intravitreally injected NMDA or glutamate mediates a rapid response and receptor mediated cell death by excitotoxicity [29]. The mode of action of extracellular HSP27 is still largely unknown. However, there are studies that have described HSP27 as an extracellular signaling molecule. Some membrane receptors for extracellular HSP could be identified. These include cluster of differentiation (CD) CD91, CD40, CD36, CD14, TLRs, and scavenger receptor-A (SR-A) [30]. In human microvascular endothelial cells, TLR3 has been identified as the receptor targeted by HSP27, which led to activation of NFκB and secretion of interleukin 8 and vascular endothelial growth factor [31]. Although not much is known about apoptotic signaling pathways related to extracellular HSP27, our investigations suggest that extracellular HSP27 can induce both intrinsic and extrinsic apoptosis, since mRNA levels of both *Casp8* and *Casp9* were elevated. Possibly, the injection of HSP7 also led to a TLR binding. Depending on the TLR, different signaling pathways can lead to apoptosis. For example, the binding to TLR2 leads to the recruitment of caspase 8 via several signaling pathways, which in turn leads to cleaved caspase 3 inducing apoptosis [32]. TLR3-initiated apoptosis seems to be mainly triggered by caspase 8 and carried out by caspase 3 [33]. It is also possible that the extrinsic and intrinsic apoptosis signaling pathways influence each other. Activated caspase 8 is able to cleave BH3 interacting-domain death agonist (BID), a pro-apoptotic member of the Bcl-2 family. After cleavage, shortened BID translocates from the cytoplasm into mitochondria and promotes cytochrome c release, which leads to caspase 9 activation, and finally induces apoptosis [34]. In addition, shortened BID and the simultaneous initiation of the intrinsic and extrinsic apoptosis pathway was detected in the glaucomatous eyes of an experimental rat glaucoma model [35]. This indicates that both apoptotic signaling pathways might be activated in glaucoma.

**Figure 8:**
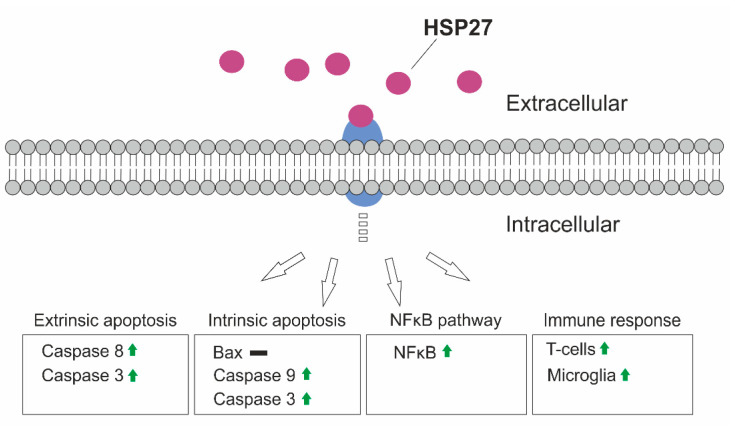
Summary of the degenerative mechanisms. HSP27 might act as an extracellular signaling molecule and binds to a receptor. As a result, intrinsic and extrinsic apoptotic mechanisms are activated. The NFκB pathway could also be activated. In addition, an increase in microglia/macrophages as well as T-cells can be observed in the retina.

In this intravitreal HSP27 injection model, neuronal degeneration also seems to occur by activation of intrinsic and extrinsic apoptosis pathways. The HSP27 injection led to an increased mRNA expression of *Casp 3*, *9*, and *8*. In addition, a higher number of cleaved caspase 3^+^ cells, was demonstrated in the optic nerve. Other apoptosis-regulating factors such as Bax, p38 (*Mapk14*), protein kinase D (*Prkd1*), and serine/threonine kinase 1 (*Akt1*) were not altered at the time of investigation. The activation of caspases via a yet unknown receptor, therefore, is the likely major trigger for retinal degeneration in this intravitreal HSP27 injection model (Figure 8).

### 3.3. NFκB Activation and Induction of an Immune Response

In addition to the increased level of caspases, immunological components after HSP27 injection were also investigated. It could be demonstrated that, regarding mRNA and protein level, more retinal NFκB, a specific transcription factor, which is important for the regulation of immune response, was detected. Therefore, HSP27 injection could also induce NFκB activation. As mentioned before, this could also be induced by an interaction between HSP27 and TLR, since NFκB activation by TLR bonding was already detected in other models [9,31]. In any case, the activation of NFκB leads to NFκB migration into the cell nucleus, where it induces a variability of proinflammatory processes [36]. One result of this altered transcription may be the observed activation of microglia/macrophages. NFκB is considered to be a key transcription factor of M1 macrophages and is required for the induction of a large number of inflammatory genes, including those encoding TNF-α, IL-1β, and IL-6 [37]. This could cause more microglia/macrophages to proliferate and activate in the retina. The observed response of microglia/macrophages in the retina could additionally contribute to neuronal damage. The negative characteristics of an uncontrolled microglial response were already observed in many other glaucoma models, this included the DBA/2J mouse model, where a microglial reaction was observed before the neuronal damage occurred [38]. In addition, in a model of ocular hypertension, microglia reactions in the retina, optic nerve, and optic tract have been noted [39].

Another interesting function of NFκB is the regulation of inflammatory response. NFκB can mediate the induction of various proinflammatory genes, and also regulate the activation, differentiation, and effector function of inflammatory T-cells [40,41]. In recent years, it has been speculated whether elevated HSP27 levels induce an immune response in glaucoma. Tezel et al., twenty years ago, postulated that long-lasting HSP expression could promote an immune response [11]. Examination of CD3^+^ T-cells demonstrated very few specific signal bindings in our study, but cell counts demonstrated more T-cells in HSP27 retinae. This could indicate that if the extracellular concentration of HSP27 is increased and lasts longer, an immune response against HSP27 may occur. Speculations about this have been published earlier, as glaucoma patients displayed elevated antibody titers against HSP27 and other HSPs [42,43,44]. Nevertheless, further studies are needed to pursue and possibly confirm this theory.

It should also be noted that the observed degenerative mechanisms appear to be in the retina rather than in the optic nerve. Activation of the NFkB signaling pathway, as well as microglia/macrophage response and T-cell recruitment, were not observed in the optic nerve. It is possible that the injected HSP27 is degraded so quickly that it has no effect on cells located further away in the optic nerve. The increased caspase 3 signal and the degeneration of the neurofilament observed after 21 days could be a consequence of the degenerative mechanisms in the retina [17]. It should be noted that only one HSP27 dosage (0.4 µg) was tested. Since we already used 0.4 µg HSP27 in a previous study, this was necessary for reasons of comparability [17]. We were previously able to observe neuronal degeneration 21 days after intravitreal HSP27 injection, in this study, we detected an activation of the intrinsic and extrinsic apoptosis pathway. Still, the investigation of higher HSP27 concentrations and a possible dose-dependent effect are aspects that still need to be analyzed in further studies.

## 4. Materials and Methods

### 4.1. Animals

All experiments concerning animals were carried out in accordance with the ARVO Statement for Use of Animals in Research and approved by the animal care committee of North Rhine-Westphalia (Germany, approval number 84-02.04.2013.A442, permission date 11 February 2014). In our study, 26 male Wistar rats (376–400 g, Charles River, Sulzfeld, Germany) were used. All animals were kept on a light-dark cycle (12 h/12 h) and an unlimited access to food and water was guaranteed. Health checks were performed regularly.

### 4.2. Intravitreal HSP27 Injection

The intravitreal injections of HSP27 or PBS were performed, as previously described [26]. Briefly, rats were anesthetized (ketamine, 50 mg/mL, Ratiopharm (Ulm, Germany) and xylazine, 2% (Bayer Health Care, Leverkusen, Germany). A mydriatic (Tropicamid 5 mg/mL, Stulln, Germany) was used to dilate the pupils. To perform the intravitreal injection, eyes were treated with a local anesthetic (Conjuncain 4 mg/mL, Bausch & Lomb, Berlin, Germany). With a 32-gauge needle (Hamilton, Reno, NV, USA), 2 µL of 0.2 µg/µL HSP27 solution (dose per eye 0.4 µg, AtGen, Yatap-dong, Korea) or 2 µL PBS (Biochrom GmbH, Berlin, Germany) were injected under a stereomicroscope (Zeiss, Oberkochen, Germany) in one eye per animal. To avoid damage to the eye an antibiotic ointment (Floxal, Bausch & Lomb, Berlin, Germany) was applied afterwards. The corresponding eye remained untreated.

### 4.3. Preparation of Retina and Optic Nerve

After 14 days, eyes (*n* = 13/group) and optic nerves (*n* = 8/group) were explanted. Five retinae were frozen directly at −80 °C for later quantitative real-time PCR (RT-qPCR) analysis. The other organs were prepared for retinal cross-sections (*n* = 8/group) or longitudinal optic nerve (*n* = 8/group) sections, as previously described [17]. The organs were fixed in 4% paraformaldehyde (eyes for 1 h and optic nerves for 2 h, Merck, Burlington, MA, USA), cryo-conserved in 30% sucrose overnight, and frozen embedded in NEG-50 Tissue Tek medium (Thermo Fisher Scientific, Waltham, MA, USA). The retina cross-sections (10 µm) and longitudinal optic nerve sections (4 µm) were attached on glass slides (Superfrost Plus, Thermo Fisher Scientific), fixed in ice-cold acetone, and used for immunohistological staining.

### 4.4. Specific Immunohistological Stainings of Retina and Optic Nerve

Immunofluorescence stainings were performed on retinal cross-sections (6 sections/animal, *n* = 8/group) and longitudinal optic nerve sections (6 sections/animal, *n* = 8/group) [45]. The advantage of immunohistological staining is that in addition to a quantitative evaluation of the signals, the localization of the investigated proteins and structures can also be determined. All sections were blocked with a mixture of a 10–20% serum, 0.1–0.2% Triton X-100 (Sigma-Aldrich, St. Louis, MO, USA) and PBS (ChemCruz, Dallas, TX, USA). Sections were treated over night with primary antibodies (Table 1) diluted in the same block solution. The next day, Cy3/Alexa Fluor 555 or Alexa Fluor 488 labeled secondary antibodies (Table 1) were applied. Cell nuclei were visualized with 4′,6-diamidin-2-phenylindole (DAPI, Serva Electrophoresis, Heidelberg, Germany). Finally, the sections were covered with Shandon-mount (Thermo Fisher Scientific). Negative controls were performed by applying only the secondary antibody.

A directly labeled antibody was used for CD3 staining of T-cells. Hence, after the CD3 antibody was applied to the sections, an overnight incubation took place. On the next day, the nuclei were stained with DAPI.

Four images per retina (two peripheral and two central) and three per optic nerve (proximal, middle, and distal) were taken using a fluorescence microscope (Axio Imager M2, Carl Zeiss Microscopy) with a Zeiss objective lens (40× and 20×) [45,46] and an Axiocam HRc CCD camera (Zeiss). The recording software used was ZEN 2012. All images, except the CD3 staining, were taken at 400× magnification. For CD3 staining, the images were taken at 200× magnification, since signals were counted in all retinal layers. RBPMS^+^, NFκB^+^, CD3^+^, and cleaved caspase 3^+^ cells were counted in masked pictures if these signals matched DAPI signals. In regard to ED1, the co-localization with Iba1 was evaluated in the GCL and inner plexiform layer (IPL). Bax^+^ signals were also counted in co-localization with RBPMS^+^ signals. NFκB^+^ signals were also counted in the GCL. All cells were counted using ImageJ software (V 1.53, NIH).

To analyze the endogenous HSP27 in the retina and optic nerve, an HSP25 antibody was used, as this is the rodent homologue to human HSP27. HSP25 signals in retina and optic nerve were investigated via area analysis using an ImageJ macro [45]. For this analysis, all retina images were converted into 32-bit gray scale. After background subtraction (48.79), the lower threshold was set at 20.9 and upper threshold at 23.6. In the optic nerve, the background of 49.7 was subtracted, while the lower threshold was set at 57.7 and the upper one at 6.5.

SMI-32 labeled optic nerve neurofilaments were scored using an established scoring system ranging from 0 = intact up to 2 = destroyed, in 0.5 intervals [45]. The figures presented were created with the help of the CorelDRAW software (V: 2018, 64-bit, Corel Corporation, Ottawa, ON, Canada).

### 4.5. Quantitative Real-Time PCR Analysis of Retinal Tissue

For RNA isolation, the retinae (*n* = 5/group) were dissected from the eyes and directly frozen at −80 °C. The Gene Elute Mammalian RNA Miniprep Kit (Sigma-Aldrich) was used for RNA extraction and purification. Then, 1 µg RNA was used for reverse transcription with a cDNA synthesis kit (Thermo Fisher Scientific). The RT-qPCR experiments were performed in a PikoReal 96 real-time PCR system (Thermo Fisher Scientific) using SYBR Green. The designed oligonucleotides for RT-qPCR are presented in Table 2. The genes Actb (Actin, beta) and Ppid (Cyclophilin) served as reference genes.

### 4.6. Statistical Analysis

The immunohistological cell counts of the native group were set to 100% and compared to the HSP27 and the PBS group. The immunohistological data were analyzed using Statistica software (Version 13.3, Dell, Round Rock, TX, USA) using one-way ANOVA followed by Tukey post-hoc test and presented as mean ± standard error mean (SEM) with * *p* < 0.05, ** *p* < 0.01, and *** *p* < 0.001. In regard to RT-qPCR analyses, the relative expression values are presented as median ± quartile + minimum/ maximum. The analysis of relative expression was performed by the Pair Wise Fixed Reallocation Randomization Test © using REST © software (Qiagen, Hilden, Germany).

## 5. Conclusions

In summary, intravitreally injected HSP27 affected primarily the retina. There, it led to an increased level of caspases 3, 8, and 9 after 14 days, which suggested the involvement of the intrinsic and extrinsic apoptosis pathway. In addition, an activation of retinal NFκB was noted, which induced a probable reaction of microglia/macrophages. First indications of an immune response were also observed via an increased number of retinal T-cells (Figure 8). The damage in the optic nerve seemed to be mostly a consequence of retinal damage, as hardly any degenerative mechanisms had been observed in the optic nerve at the analyzed point in time.

## Figures and Tables

**Table 1:** Primary and secondary antibodies used for immunohistochemistry.

Primary Antibodies			Secondary Antibodies		
Name and host	Company	Dilution	Name	Company	Dilution
**Retinal Cross-sections**					
Bax (rabbit)	Abcam	1:100	Donkey anti-rabbit Alexa Fluor 555	Abcam	1:400
CD3-FITC (directly labeled antibody, mouse)	BD Biosciences	1:1000	−	−	−
ED1 (mouse)	Millipore	1:250	Goat anti-mouse Alexa Fluor 488	Invitrogen	1:400
GFAP (chicken)	Millipore	1:400	Donkey anti-chicken Cy3	Millipore	1:500
HSP25 (rabbit)	Enzo Life Sciences	1:100	Donkey anti-rabbit Alexa Fluor 488	Jackson ImmunoResearch	1:500
Iba1 (rabbit)	Wako	1:500	Goat anti-rabbit Cy3	Linaris	1:300
NeuN (chicken)	Millipore	1:500	Donkey anti-chicken Cy3	Millipore	1:500
NFκB (rabbit)	Santa Cruz	1:500	Goat anti-rabbit Alexa Fluor 488	Invitrogen	1:500
RBPMS (guinea pig)	Millipore	1:250	Donkey anti-guinea pig Alexa 488	Jackson ImmunoResearch	1:400
**Longitudinal Optic Nerve** **Sections**					
CD3-FITC (directly labeled antibody, mouse)	BD Biosciences	1:100	−	−	−
Cl. caspase3 (rabbit)	Sigma-Aldrich	1:300	Donkey anti-rabbit Alexa Fluor 555	Invitrogen	1:400
ED1 (mouse)	Millipore	1:200	Goat anti-mouse Alexa Fluor 555	Invitrogen	1:500
HSP25 (rabbit)	Enzo Life Sciences	1:100	Donkey anti-rabbit Alexa Fluor 488	Jackson ImmunoResearch	1:500
Iba1 (rabbit)	Wako	1:400	Goat anti-rabbit Alexa Fluor 488	Invitrogen	1:500
SMI-32 (mouse)	Biolegend	1:2000	Goat anti-mouse Alexa Fluor 488	Invitrogen	1:400

**Table 2:** List of oligonucleotides used for mRNA expression analysis in retinae.

Gene	Primer Sequence	GenBank Accession Number
*Actb-F*	cccgcgagtacaaccttct	NM_031144.3
*Actb-R*	cgtcatccatggcgaact	
*Akt1-F*	gacgtagccattgtgaaggag	NM_033230.2
*Akt1-R*	ccatcattcttgaggaggaagt	
*Casp3-F*	ccgacttcctgtatgcttactcta	NM_012922.2
*Casp3-R*	catgacccgtcccttgaa	
*Casp8-F*	agagcctgagggaaagatgtc	NM_022277.1
*Casp8-R*	tcacatcatagttcacgccagt	
*Casp9-F*	cgtggtggtcatcctctctc	NM_031632.1
*Casp9-R*	gagcatccatctgtgccata	
*Hspb1-F*	gaggagctcacagttaagaccaa	NM_031970.4
*Hspb1-R*	ttcatcctgcctttcttcgt	
*Hspb70-F*	catatccaatatctttgaggtgga	XM_017601842.1
*Hspb70-R*	tggggaagacttcacagtca	
*Hsp90aa1-F*	gggagctcatttccaactcc	NM_175761.2
*Hsp90aa1-R*	gggttcggtcttgcttgtt	
*Mapk14-F*	gaacttcgcaaatgtatttattggt	NM_031020.2
*Mapk14-R*	cgagtccaaaaccagcatc	
*Nfκb-F*	ctggcagctcttctcaaagc	NM_001276711.1
*Nfκb-R*	caggtcatagagaggctcaa	
*Pou4f1-F*	ctggccaacctcaagatcc	XM_008770931
*Pou4f1-R*	cgtgagcgactcgaacct	
*Ppid-F*	tgctggaccaaacacaaatg	M19553.1
*Ppid-R*	cttcccaaagaccacatgct	
*Prkd1-F*	tgctccttcaggactcctct	NM_001276715.1
*Prkd1-R*	gaagccacattcagggaact	
